# Kreasote in Diphtheria

**Published:** 1866-04

**Authors:** J. J. Knott

**Affiliations:** Griffin, Georgia


Medical and Surgical Journal.

NEW SERIES.

Vol. VIIB APRIL, 1866. No. 2.

### ORIGINAL COMMUNICATIONS.

*Kreasote in Diphtheria.*—By J. J. Knott, M. D., of Griffin, Georgia.

Owing to the speedy action and happy results following the use of Kreasote in Diphtheria, coming under my observation, I am induced to give, in a short article, the manner in which it has been employed by me during the past three years, in military and civil practice.

In the year 1863, while in charge of the Small Pox Hospital for Longstreet's Corps, Army of Northern Va., diphtheria prevailed to such alarming extent, as a sequel of that loathsome disease, variola, and the mortality was so great in the cases under my care, that I was induced to look out for some more useful mode of treatment than had been used in its management previously. Regarding the disease, in the earlier periods, as local in its character, and of a septic tendency, I determined to test the virtues of this powerful antiseptic, local alternative, and styptic.

The first case in which I used it, was a very malignant one; so much so, that at one time I had almost despaired of his recovery. The following formula gives about the strength in which the remedy was applied to the parts affected:

℞ Kreasote ʒji.

Aqua Font. ℥ji.

Pulv. Acacia QS.

A sponge saturated with the Kreasote thus suspended in mucilage, was applied to the parts where the pseudo membranous exudations were exhibited, early in the afternoon. In a few hours another application was made, and, without further treatment, all the more violent symptoms disappeared during the night. On the following morning my patient seemed so much relieved that further treatment with the remedy was unnecessary.

Continuing this application in the treatment of subsequent cases, I lost no more cases from this disease.

After my return to the 53d Georgia Regiment, as Surgeon of that command, so successful was this mode of treating diphtheria, that it rarely, if ever, became necessary to send a case of the disease to the General Hospital, although several severe cases occurred in the regiment.

Since my return from the army, I have adopted this treatment in several cases of a decidedly diphtheritic character, in all of which much benefit was derived, and in one case particularly, which I distinctly remember to have been relieved almost entirely by one application, after suffering for a week under the ordinary treatment.

What has been said in this short article is intended, at least, to call attention to an important remedial agent in the treatment of a sometimes very troublesome and disagreeable disease.

